# Marriage and risk of dementia: systematic review and meta-analysis of observational studies

**DOI:** 10.1136/jnnp-2017-316274

**Published:** 2017-11-28

**Authors:** Andrew Sommerlad, Joshua Ruegger, Archana Singh-Manoux, Glyn Lewis, Gill Livingston

**Affiliations:** 1 Division of Psychiatry, University College London, London, UK; 2 Camden and Islington NHS Foundation Trust, London, UK; 3 INSERM U 1018, Epidemiology of Ageing and Age-Related Diseases, Villejuif, France; 4 Department of Epidemiology and Public Health, University College London, London, UK

## Abstract

**Background:**

Being married is associated with healthier lifestyle behaviours and lower mortality and may reduce risk for dementia due to life-course factors. We conducted a systematic review and meta-analysis of studies of the association between marital status and the risk of developing dementia.

**Methods:**

We searched medical databases and contacted experts in the field for relevant studies reporting the relationship, adjusted for age and sex, between marital status and dementia. We rated methodological quality and conducted random-effects meta-analyses to summarise relative risks of being widowed, divorced or lifelong single, compared with being married. Secondary stratified analyses with meta-regression examined the impact of clinical and social context and study methodology on findings.

**Results:**

We included 15 studies with 812 047 participants. Compared with those who are married, lifelong single (relative risk=1.42 (95% CI 1.07 to 1.90)) and widowed (1.20 (1.02 to 1.41)) people have elevated risk of dementia. We did not find an association in divorced people.

Further analyses showed that less education partially confounds the risk in widowhood and worse physical health the elevated risk in lifelong single people. Compared with studies that used clinical registers for ascertaining dementia diagnoses, those which clinically examined all participants found higher risk for being unmarried.

**Conclusions:**

Being married is associated with reduced risk of dementia than widowed and lifelong single people, who are also underdiagnosed in routine clinical practice. Dementia prevention in unmarried people should focus on education and physical health and should consider the possible effect of social engagement as a modifiable risk factor.

## Introduction

The rising number of people living with dementia^1^ makes it the current global public health priority,^2^ and there is a pressing need to identify modifiable risk factors. Although there are more people with dementia overall, there has been a small decline in the age-specific incidence of dementia in many developed countries^3 4^ over the past two decades suggesting that differential lifetime exposure to risk factors in successive generations affects their dementia risk.^4^

Marital status has potential to affect dementia risk by increasing daily social interaction. This may improve cognitive reserve, meaning that an individual has a greater ability to cope with neuropathological damage by using compensatory cognitive approaches from a physically more resilient brain to maintain cognitive ability and daily function.^5^ Marriage may result in more frequent social contact, which is associated with reduced dementia risk,^6^ and reduced harmful lifestyle behaviours.^7 8^ Bereavement or divorce in people who had been married may promote dementia development through stress, which is pathogenic^9^ and associated with increased dementia risk.^10^ Being unmarried is associated with adverse health behaviours^7^ and a range of poorer health outcomes. A meta-analysis of observational studies found lower mortality for married than unmarried people^11^; health of unmarried Americans is worse than that of married people^8^; being married is related to improved cancer survival^12^; and widowhood is associated with disability in older people.^13^

In this study, we aim to synthesise evidence from published studies examining the effect of marital status (married/cohabiting, widowed, divorced/separated and lifelong single) on dementia incidence and the extent to which this risk is modified by sociodemographic factors, study design and methodological quality of the study. We hypothesise that married people are at lower risk of developing dementia compared with unmarried people and that previously married people are at lower risk than those who have been lifelong single.

## Methods

### Search strategy

We searched Embase, MEDLINE and PsycInfo databases from their inception to 5 December 2016. Our search terms (online supplementary table 1) identified papers whose titles, abstracts or keywords included terms encompassing marital status and dementia, and we used the Scottish Intercollegiate Guidelines Network filters for observational studies (http://www.sign.ac.uk/methodology/filters.html). We searched references of included studies and systematic reviews and contacted two experts in this field aiming to identify additional studies.

10.1136/jnnp-2017-316274.supp1 Supplementary material 1

### Inclusion criteria

A study was included if:

- it used a prospective or retrospective cohort, case–control or cross-sectional study design
- it reported quantitative data measuring the relationship between dementia and marital status or partner/spouse presence
- it presented results of analyses that were adjusted for age and sex; we contacted authors of studies who reported unadjusted results and included new adjusted data if provided
- marital status was measured and reported separately from other aspects of social network, for example, contact with other family
- the sample consisted of at least 50% of individuals aged 65 years or over at time of dementia ascertainment, or if a younger population was sampled, a study was included if it presented stratified results for an over-65 population
- the sample was derived from a general community-dwelling population. For cohort studies, participants had to be screened for dementia at baseline and prevalent dementia cases excluded.
- it was a published research paper or dissertation; when we found relevant conference abstracts, we contacted the author for details of any eligible published research
- it was published in English.

When two studies reported different analyses of cohort studies, so to avoid duplication, we used only the analysis that had a longer follow-up duration.

### Data extraction

One researcher (AS) screened the abstracts of all studies to identify those potentially meeting the inclusion criteria and reviewed full-text articles to confirm eligibility. A second researcher (JR) reviewed a random sample of 10% of the studies to assess agreement and reviewed all included studies to approve eligibility. We used a standardised form (online supplementary table 2) to extract data for evidence synthesis. Extracted information included results and information for the assessment of the risk of bias.

In the one study^14^ that used lifelong single people as the reference group, we inverted the ORs, and for this study and another,^15^ we calculated CIs based on raw published data.^16^ Where marital status categories had been combined (eg, divorced and single people) or results for dementia subtypes rather than all-cause dementia presented, we requested additional data from study authors. We have included new data for three papers.^17–19^

We registered the study protocol prospectively in the PROSPERO register of systematic reviews (http://www.crd.york.ac.uk/PROSPERO/display_record.asp?ID=CRD42016043161).

### Quality rating

We rated methodological quality of included studies using an adapted version of the *Newcastle-Ottawa Criteria* 20 for cohort and case–control studies and the *Joanna Briggs Institute’s Checklist* 21 for cross-sectional studies. Full details are in online supplementary tables 3a–c but, in summary, these tools rated the quality of selection, measurement and comparability for all studies and gave a score for cohort and case–control studies (maximum of 9) and cross-sectional studies (maximum 6). Two researchers (AS and JR) assessed the quality of all included studies and discussed discrepancies until consensus was reached.

### Statistical analysis

We provide a narrative synthesis of findings from included studies and have pooled results where studies have used the same measurements, calculating random-effects estimates using STATA V.14. The random-effects model allows for HRs and ORs to be incorporated into the same meta-analysis^22^ and accounts for heterogeneity between studies.^23^ All included studies provided an estimate of relative risk and CI that we used for the analysis. We measured heterogeneity between the studies using the χ^2^ test and the *I* 2 statistic and considered, a priori, that *I* 2 >50% indicated substantial heterogeneity. Where studies provided estimates of relative risk from different multivariate models, we included the result from the model with the largest number of covariates.

Our main analyses compared risk of all-cause dementia in married people to those who were widowed, divorced or lifelong single for studies that ascertained dementia diagnosis status from clinical assessment. We conducted prespecified secondary analyses. We analysed the association between marital status and risk of Alzheimer’s or vascular dementia. We conducted stratified analyses and used meta-regression^24^ to quantify the effect of various study design factors on the association between marital status and all-cause mortality: (1) dementia case ascertainment method: clinical assessment of study participants versus clinical register data; (2) study type: cohort versus other studies; (3) study quality rating; and (4) time period of study conduct, based on mean year of birth of study participants.

We assessed the effect of confounder adjustment on the relative risk using stratified analyses of studies that adjusted only for age and sex versus studies that additionally adjusted for education or baseline cognition versus studies that additionally adjusted for physical health. We assessed for evidence of publication bias using funnel plots and Egger’s weighted regression method.^25^

## Results

**Figure 1:**
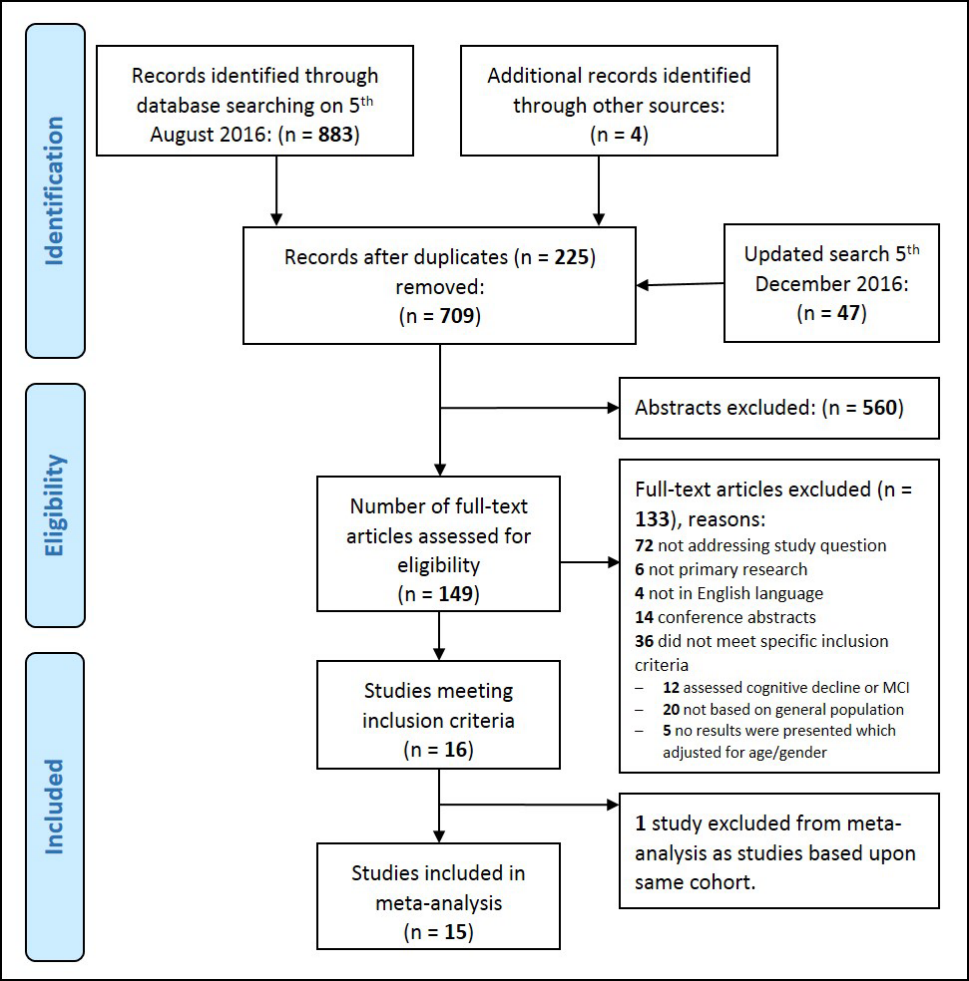
PRISMA diagram of study identification and selection. PRISMA, Preferred Reporting Items for Systematic Review and Meta-Analysis.

The Preferred Reporting Items for Systematic Review and Meta-Analysis (PRISMA) diagram (figure 1) shows our search results and reasons for study exclusion. Sixteen studies fulfilled our inclusion criteria, but we excluded one publication^26^ from our meta-analysis as it reported data from the same cohort as another study^27^ but with shorter follow-up. The 15 studies in our analyses included 812 047 people, of whom 29 610 had any form of dementia. Of these, 61 012 had a clinical assessment for dementia and 751 035 had dementia status ascertained from clinical records.

MCI, Mild cognitive impairment.

Table 1 describes key study characteristics. Nine were cohort studies,^17–19 27–32^ two case–control^14 15^ and four cross-sectional.^33–36^ Eight included studies were set in European countries, four in Asia, two from USA and one from Brazil. The mean year of birth of study participants ranged from 1897 to 1939. Studies typically measured marital status at study inception (mean age 72.8 (SD 7.2) years.) In the cohort studies, the duration of follow-up before dementia assessment was 3 to 20.9 (mean 8.5, SD 5.5) years.

**Table 1:** Characteristics of included studies

Study (first author and year of publication)	Study design	Country	Number of participants	Mean year of birth	Mean age at marital status evaluation (years)	Baseline marital status of participants (%)				Mean/range of follow-up (years)	Method of dementia case ascertainment	Quality rating
						Married	Widowed	Divorced	Lifelong single			
Amieva 2010^27^	Cohort	France	2089	1914	74	60.7	32.5	2.7	4.2	5–15	Clinical assessment	5
Arai 2004^28^	Cohort	Japan	853	1929	69	71	29 (unmarried)			5	Clinical assessment	3
Bae 2014^17^	Cohort	S Korea	359	1936	72	70.2	29.8	0	0	3.5	Clinical assessment	3
Bickel 1994^18^	Cohort	Germany	331	1918	74	42.4	47.5	3.8	6.4	7–8	Clinical assessment	5
Fratiglioni 2000^19^	Cohort	Sweden	1368	1905	82	27.8	45.4	5.9	20.9	3	Clinical assessment	6
Håkansson 2009^29^	Cohort	Sweden	2000	1926	51	80.1	7.8	4.4	7.8	20.9	Clinical assessment	8
Hatch 2013^30^	Cohort	USA	5092	1920	75	65.9	29.9	4.1	N/A	12	Clinical assessment	8
Sundström 2014^31^	Cohort	Sweden	1677	1919	75	57.6	14.2	5.7	32.6	8.6	Clinical assessment	7
Sundström 2016^32^	Cohort	Sweden	7 50 129	1928	69	64.9	8.4	16.0	10.8	6	Clinical register/death register	9
Beard 1992^15^	Case-control	USA	482	1897	80	28.8	48.0	5.4	17.8	N/A	Secondary care clinical register	3
Seidler 2003^14^	Case-control	Germany	424	1924	77	78.5	11.1	3.8	6.6	N/A	General practice clinical register	2
Correa Ribeiro 2013^33^	Cross-sectional	Brazil	683	1931	78	41.6	40.8	7.5	10.1	N/A	Clinical assessment	3
Fan 2015^34^	Cross-sectional	Taiwan	10 432	1936	76	64.2	31.0	4.8 (Div/single)		N/A	Clinical assessment	4
Guaita 2015^35^	Cross-sectional	Italy	1321	1939	72	67.1	24.6	2.2	6.1	N/A	Clinical assessment	4
Zhang 2006^36^	Cross-sectional	China	34 807	1929	68	77.4	20.8	1.6 (Div/single)		N/A	Clinical assessment	4

N/A, Not applicable

Married people accounted for between 27.8% and 80.1% of the sample (widowed=7.8% to 48.0%, divorced=0% to 16%, lifelong single=0% to 32.6%). Two studies^34 36^ combined divorced and lifelong single people (6.1% and 10.1%). The mean methodological quality score for the cohort studies was 5.4/9, 2/9 for case–control studies and 3.8/6 for cross-sectional studies. Full details of methodological assessment are in online supplementary tables 3a–c. All included cohort studies analysed complete cases, excluding participants who had withdrawn from study.

Marital status was, in all but two of the cohort studies^30 32^ which used registry data, reported by the participant or a close informant. No studies provided further details about this assessment nor was there any information on duration of exposure to a particular marital status category. In one cohort study,^32^ marital status was ascertained from a Swedish central population register, and in another cohort,^30^ a marriage registry was used to confirm marital status. For the two case–control studies, those with dementia (or, if incapable of answering, an informant) were asked about their marital status at age 30 and 50 years and 10 years prior to interview^14^ or at time of diagnosis.^15^

All but three of the studies clinically examined all participants for ascertaining diagnostic status (outcome). The other studies^14 15 32^ ascertained diagnostic status from routine clinical registers and, for one of these studies,^32^ death registers. Except for the cohort study^32^ that exclusively used register data, none reported whether they ascertained dementia status from death registers. The clinical examination used in the majority of studies was a staged approach: a screening phase followed by a more detailed neuropsychological and functional assessment and an expert consensus panel to establish diagnostic status.

### Main meta-analysis: widowed, divorced or lifelong single versus married people and risk of all-cause dementia

**Figure 2:**
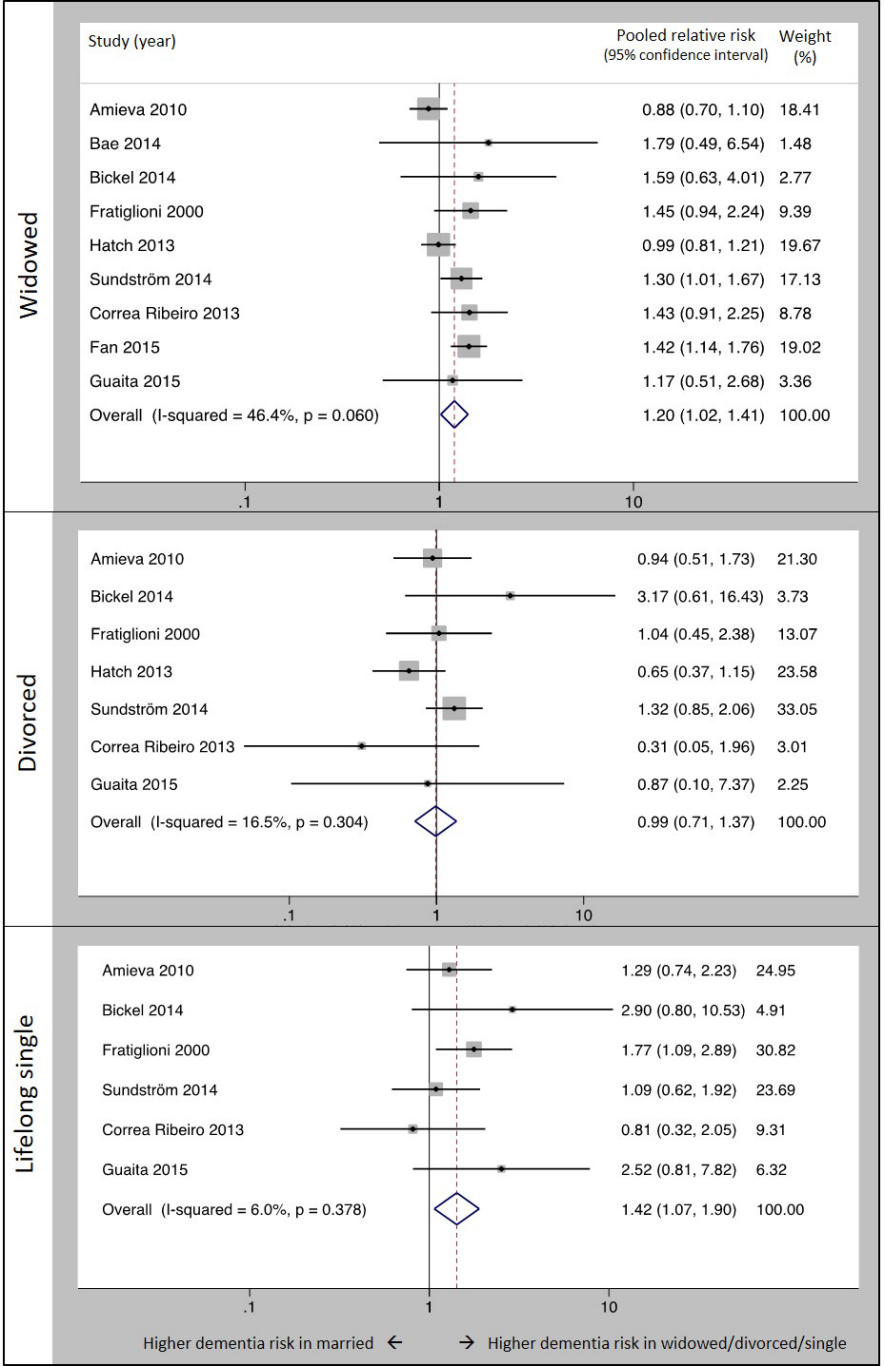
Forest plot showing pooled relative risk of dementia in widowed, divorced and lifelong single people versus married people when dementia was ascertained by clinical examination. Notes: figures are based on random-effects meta-analysis; included studies ascertained dementia diagnostic status using a clinical examination of study participants.

We pooled risk estimates from studies that evaluated the risk of all-cause dementia according to marital status category, with dementia case ascertainment based on clinical examination (figure 2). Nine studies analysed the risk of all-cause dementia in widowed versus married people and we found that in widowed, compared with married, people, the relative risk of dementia=1.20 (95% CI 1.02 to 1.41). The relative risk for divorced versus married people from seven studies=0.99 (0.71 to 1.37) and for the six studies that analysed dementia risk for lifelong single people, RR=1.42 (1.07 to 1.90).

### Secondary analyses

#### Widowed, divorced or lifelong single versus married people and risk of Alzheimer’s disease and vascular dementia

Fewer studies examined the risk of dementia subtypes according to marital status. Eight^14 15 17 27 29 30 35 36^ examined the risk of Alzheimer’s disease (1891 cases) in widowed versus married people and found a pooled relative risk of 1.24 (0.97 to 1.60). The risk of Alzheimer’s disease in five^14 15 27 30 35^ studies of divorced (0.89 (0.58 to 1.36)) and three^15 27 35^ of lifelong single (1.07 (0.75 to 1.52)) people was not different to that of married people. For vascular dementia (372 cases), no effect of marital status on dementia risk was found in pooled estimates from the three studies^14 35 36^ that examined the risk for widowed versus married people (pooled RR=0.90 (0.40 to 2.04)) or the two^14 35^ studies that examined risk in lifelong single people versus married (2.66 (0.85 to 8.28)). Only one study^14^ compared the risk of vascular dementia in divorced and married people and found no difference.

#### Widowed, divorced or lifelong single versus married people and risk of all-cause dementia stratified by sex

Two studies (online supplementary table 2) analysed the relationship between marital status and dementia separately for men and women. For one study,^32^ the outcome was all-cause dementia, and for the other,^15^ it was Alzheimer’s disease so meta-analysis was not possible. Neither study found any difference between men and women in the association of marital status and dementia.

#### Impact of study design on association between marital status and all-cause dementia

##### Widowed, divorced or lifelong single versus married people and risk of all-cause dementia stratified by case ascertainment method

There was evidence that the method of dementia case ascertainment affected the risk estimates (table 2). Studies using clinical examination for dementia ascertainment produced higher pooled estimates for the effect of being widowed (1.20 (1.02 to 1.41) versus 1.12 (1.07 to 1.18)) or lifelong single (1.42 (1.07 to 1.90) versus 1.23 (1.17 to 1.29)), and this difference nearly reached significance for the comparison of single and married people (p=0.06). The risk of dementia for divorced compared with married people was slightly lower but neither risk estimate was significant.

**Table 2:** Meta-regression of the risk of all-cause dementia according to marital status, stratified by study time period, case ascertainment methodology, study type and study quality

		Widowed versus married		Divorced versus married		Lifelong single versus married	
		Stratified analysis: Relative risk (95% CI) Number of studies	Meta-regression coefficient (95% CI) p value	Stratified analysis: Relative risk (95% CI) Number of studies	Meta-regression coefficient (95% CI) p-value	Stratified analysis: Relative risk (95% CI) Number of studies	Meta-regression coefficient (95% CI) p value
Method of case ascertainment	Clinical assessment	1.20 (1.02 to 1.41) n=9	b=−0.06 (−0.18 to 0.05) p=0.29	0.99 (0.71 to 1.37) n=7	b=0.34 (0.06 to 0.62) p=0.02	1.42 (1.07 to 1.90) n=6	b=−0.27 (−0.55 to 0.01) p=0.06
	Clinical registers	1.12 (1.07 to 1.18) n=2		1.11 (0.52 to 2.38) n=2		1.23 (1.17 to 1.29) n=2	
Study type	Cohort	1.10 (1.05 to 1.28) n=7	b=0.28 (0.09 to 0.46) p=0.004	1.16 (0.87 to 1.55) n=6	b=−0.83 (−1.69 to 0.03) p=0.06	1.24 (1.17 to 1.30) n=5	b=0.08 (−0.45 to 0.62) p=0.76
	Case–control/cross-sectional	1.39 (1.16 to 1.67) n=4		0.55 (0.23 to 1.31) n=3		1.21 (0.67 to 2.18) n=3	
Global quality score	Higher quality ≥6	1.13 (1.02 to 1.31) n=4	b=0.08 (-0.06 to 0.23) p=0.27	1.16 (0.83 to 1.62) n=4	b=−0.40 (-0.88 to 0.08) p=0.10	1.26 (1.09 to 1.45) n=3	b=0.20 (-0.17 to 0.57) p=0.29
	Lower quality <6	1.22 (0.96 to 1.54) n=7		0.88 (0.54 to 1.44) n=5		1.33 (0.92 to 1.92) n=5	
	Increase in quality by one point	b=−0.04 (−0.08 to −0.002) p=0.04 n=11		b=0.12 (0.01 to 0.24) p=0.04 n=9		b=−0.05 (−0.13 to 0.03) p=0.21 n=8	
Time period	Mean DoB before 1927	1.11 (0.93 to 1.31) n=6	b=0.15 (−0.14 to 0.43) p=0.32	0.98 (0.71 to 1.37) n=6	b=0.35 (0.08 to 0.63) p=0.01	1.40 (1.06 to 1.85) n=5	b=−0.22 (−0.50 to 0.06) p=0.13
	Mean DoB after 1927	1.23 (1.06 to 1.43) n=5		1.08 (0.50 to 2.35) n=3		1.24 (0.94 to 1.62) n=3	
	Mean year of birth 10 years later	b=0.08 (−0.08 to 0.23) p=0.34 n=11		b=0.24 (0.01 to 0.47) p=0.04 n=9		b=−0.15 (−0.33 to 0.02) p=0.09 n=8	

Figures are based on random-effects meta-analysis.

DoB, date of birth.

##### Widowed, divorced or lifelong single versus married people and risk of all-cause dementia stratified by study type

The pooled risk estimate (table 2) for dementia in widowed versus married people was lower (meta-regression: p=0.004) from the seven cohort studies^17–19 27 30–32^ (1.10 (1.05 to 1.28)) than the four cross-sectional or case–control studies^14 33 34 36^ (1.39 (1.16 to 1.67)) that examined this association. There were no differences between cohort and other studies in pooled estimates of dementia risk in lifelong single versus married people or divorced versus married people.

##### Widowed, divorced or lifelong single versus married people and risk of all-cause dementia stratified by study quality

Stratified analyses of higher versus lower quality studies and meta-regression analysis of the effect of study quality on risk estimates found no effect of study quality on relative risk for widowed or lifelong single people. The four higher quality studies^19 30–32^ produced a slightly increased risk for divorced people than the five lower quality studies^14 18 27 33 35^ but in neither strata was divorce related to dementia risk.

##### Widowed, divorced or lifelong single versus married people and risk of all-cause dementia by time period

Meta-regression analysis suggested that the relative risk of dementia in divorced people increased by 24% (95% CI 1% to 47%) for studies of participants born 10 years later (table 2), although risk remained non-significant when comparing the newer and older studies. There was some evidence that time period modified the effect of being lifelong single on risk of dementia: the risk of dementia in single people was 15% lower (9% CI 33% lower to 2% higher) for every 10 years later that participants were born. In the oldest studies (participants born on average before 1927), the risk of dementia in lifelong single versus married people was 1.40 (1.06 to 1.85) and for the most recent studies (of people born after 1927), the risk was 1.24 (0.94 to 1.62). No significant modifying effect of time period was found for the risk of dementia in widowed people.

#### Effect of covariate adjustment on risk estimates

For dementia risk in widowed versus married people, the pooled risk estimates (table 3) from the three studies^17 18 31^ that adjusted only for age and sex (1.33 (1.05 to 1.69)) was higher than the five studies^14 19 30 33 35^ that adjusted additionally for education or baseline cognitive function (1.12 (0.95 to 1.31)). No further attenuation of the effect was found in three studies^27 32 34^ that additionally adjusted for physical health (1.12 (0.92 to 1.37)).

**Table 3:** Meta-analyses of the risk of all cause dementia according to marital status stratified by covariate adjustment.

	Widowed versus married		Divorced versus married		Lifelong single versus married	
	Relative risk (95% CI) p value	Number of studies Heterogeneity statistic	Relative risk (95% CI) p value	Number of studies Heterogeneity statistic	Relative risk (95% CI) p value	Number of studies Heterogeneity statistic
Studies adjusted for age and sex	1.33 (1.05 to 1.69) p=0.02	n=3 I^2^=0%	1.41 (0.90 to 2.21) p=0.14	n=2 I^2^=1.5%	1.49 (0.61 to 3.63) p=0.38	n=2 I^2^=46.1%
Studies adjusted for age, sex and education	1.12 (0.95 to 1.31) p=0.19	n=5 I^2^=0%	0.70 (0.47 to 1.06) p=0.10	n=5 I^2^=0%	1.45 (0.97 to 2.19) p=0.005	n=4 I^2^=14.6%
Studies adjusted for age, sex, education and physical health	1.12 (0.92 to 1.37) p=0.26	n=3 I^2^=77.8%	1.30 (0.93 to 1.81) p=0.12	n=2 I^2^=42.5%	1.23 (1.17 to 1.29) p=0.36	n=2 I^2^=0%

Figures are based on random-effects meta-analysis.

For lifelong single people, the risk estimate for dementia was not affected by adjustment for education, but the relative risk of dementia in single versus married people fell from 1.45 (0.97 to 2.19) to 1.23 (1.17 to 1.29) in studies that adjusted for physical health.

#### Publication bias

In funnel plots (online supplementary figure 1), there was no clear evidence of asymmetry suggesting publication bias. Weighted regression (Egger) test indicated that there was unlikely to be publication bias in studies examining widowed (p=0.30) or lifelong single (p=0.35) people but that there may have been for studies of divorced people (p=0.04).

## Discussion

Our study summarised all accessible published evidence and found that people who are lifelong single have a 42% higher risk and that those who are widowed have a 20% higher risk of developing dementia than those who are married in studies adjusted for age and sex. We found no evidence that dementia risk in divorced people differs from married people. The reduced risk in married people persisted in sensitivity analyses, indicating the robustness of the findings. Similar direction and magnitude of effect were found for dementia subtypes, but these estimates were non-significant as these analyses had fewer participants. Study design affects estimates of dementia risk. Higher relative risk of dementia for lifelong single and widowed people was found in studies that diagnosed dementia following clinical examination of all participants than in those that ascertained diagnostic status from routinely collected data; and lower risk was found for widowed people in cohort studies than in case–control or cross-sectional studies. There is some indication that the elevated risk in lifelong single people has decreased over time, with more recent studies finding smaller associations. We find that much of the increased risk in widowed people is attenuated after adjustment for education and that confounding by physical health explains part of the increased risk of dementia in lifelong single people.

Our findings may be explained in one or more ways. First, being married may change individuals’ exposure to other protective and risk factors throughout their subsequent lifespan; this is supported by our identification of confounding factors affecting this risk and evidence showing married people to be more likely to have a healthy lifestyle. The residual increased risk for lifelong single people in studies that adjusted for age, sex, education and physical health is likely to be due to different social engagement in married and single people,^37^ which may contribute to building cognitive reserve and reducing dementia risk^6^ over the lifespan. The magnitude of effect of marital status on dementia is higher than the risk for mortality in unmarried compared with married people (RR=1.1),^11^ supporting the idea that marriage’s effect on dementia risk is more than just improving physical health and that there may a direct cognitive benefit of being married.

Second, the end of marriage through bereavement could act directly to increase dementia risk, through the detrimental effect of stress on hippocampal neurons^9^ or cognition,^10^ and this theory could explain the increased dementia risk for widowed, but not divorced, people, as studies have found widowhood to be more stressful than divorce.^38 39^ Third, developing dementia could be related to other underlying cognitive or personality traits meaning that in societies where marriage was the social norm, people with difficulties in flexibility of thought or communication and consequent smaller lifelong cognitive reserve (therefore more likely to develop dementia) may be less likely to marry. This explanation may be supported by our finding that the risk for lifelong single people is possibly reduced in more recent times. Remaining unmarried has become more common,^40 41^ and it may be that single people born in the latter half of the 20thcentury have fewer unusual cognitive and personality characteristics.

Our findings, from large populations, across numerous countries and time periods are the strongest evidence yet that married people are less likely to develop dementia. We searched the literature systematically, sought additional studies where possible by contacting authors to gain additional data where published information was insufficient and followed PRISMA guidance in the conduct and reporting of this study.^42^ The main limitations of this review relate to the methodology of included studies. We could not investigate the effect of the duration of being widowed or divorced as the included studies did not report this, and we could only investigate the impact of potential confounders that were measured and analysed in studies, limiting our investigation of potential explanations for our findings. Our findings in relation to divorced people are less robust as there were fewer divorced people in the included studies. While our search terms were thorough, supporting our belief that we identified all studies examining this relationship, we may have missed eligible studies. This is a particular risk for observational studies examining the effect of other exposures on dementia risk, which may have reported marital status as a potential covariate, although less likely for this review as we aimed to only include studies that adjusted the relationship between marital status and dementia for age and sex.

Our finding of a 42% increased risk in lifelong single people compares closely to other known dementia risk factors incorporated in National Institute for Health and Care Excellence guidelines^43^ such as physical inactivity (RR=1.4) and less education, hypertension or smoking (RR for each=1.6).^44^ Our findings support the need for further work to develop preventative approaches in these lifestyle domains and indicate this may be particularly important for the high-risk groups of widowed and lifelong single people.

We also found that routine clinical registers underestimate the risk of dementia in these groups, which is likely to be because register data has poor sensitivity for detecting dementia^45^ and unmarried people are more likely to be undiagnosed in routine practice.^46^ Diagnosing dementia in people who attend clinic alone is more difficult, due to lack of collateral information and because individuals with dementia may not complain of memory impairment,^47^ so clinicians should have a high index of suspicion for dementia in these groups.

Future research should explore the mechanism of the relationship between marital status and dementia in order to develop interventions. It should, in particular, evaluate the contribution of social contact and health behaviours; use studies with sufficient follow-up to allow exploration of premarriage cognitive characteristics; and use cohort studies with sufficient detail on the duration of marriage, widowhood or divorce to allow the exploration of a dose–response effect.
