# Supplementary material for: Marriage and risk of dementia: systematic review and meta-analysis of observational studies
Source: J Neurol Neurosurg Psychiatry. 2017 Nov 28;89(3):231–8. doi: 10.1136/jnnp-2017-316274 (PMC5869449; doi:10.1136/jnnp-2017-316274)

Supplementary material

Supplementary table 1. Search terms used

**Medline**		**exp marital status/ OR marriage.tw. OR married.tw. OR marital status.tw. OR spouse.tw**
	**AND**	exp Dementia/ **OR** dementia.tw. **OR** alzheimer*.tw
	**AND**	Epidemiologic studies/ **OR** exp case control studies/ **OR** exp cohort studies/ **OR** Case control.tw. **OR** (cohort adj (study or studies)).tw. **OR** Cohort analy$.tw. **OR** (Follow up adj (study or studies)).tw. **OR** (observational adj (study or studies)).tw. **OR** Longitudinal.tw. **OR** Retrospective.tw. **OR** Cross sectional.tw. **OR** Cross-sectional studies/
**Embase**		exp marriage/ **OR** marriage.tw. **OR** married.tw. **OR** marital status.tw. **OR** spouse.tw
	**AND**	exp Dementia/ **OR** dementia.tw. **OR** alzheimer*.tw
	**AND**	Clinical study/ **OR** Case control study **OR** Case control study **OR** Longitudinal study/ **OR** Retrospective study/ **OR** Prospective study/ **OR** Cohort analysis/ **OR** (Cohort adj (study or studies)).mp. **OR** (Case control adj (study or studies)).tw. **OR** (follow up adj (study or studies)).tw. **OR** (observational adj (study or studies)).tw. **OR** (epidemiologic$ adj (study or studies)).tw. **OR** (cross sectional adj (study or studies)).tw. **NOT** Randomized controlled trials/
**PsycINFO**		exp marriage/ **OR** marriage.tw. **OR** married.tw. **OR** marital status.tw. **OR** spouse.tw
	**AND**	exp dementia/ **OR** dementia.mp. **OR** Alzheimer.mp
	**AND**	exp Longitudinal studies/ **OR** cohort.mp **OR** prospective.mp **OR** longitudinal.mp **OR** retrospective.mp **OR** ((case* adj5 control*) **or** (case adj3 comparison*) **or** case-comparison **or** control group*).ti,ab.id. **NOT** "literature review".md

Supplementary table 2. Full data extracted from cohort, case-control and cross-sectional studies

Study	Recruitment source and population at start (response rate)<br><br>Mean population age at baseline	n of participant at study inception<br><br>n of cases at follow-up	Mean/ range of years follow-up	n un-explained loss to follow-up / missing data %	Measurements of marital status (%)<br>At what age, and approx. what year was marital status recorded?	Analysis adjusted for:	Statistical model used	Outcome How was dementia assessed?	Results	Adjusted results (95% Confidence interval)	Un-adjusted results
**COHORT**											
Amieva 2010	PAQUID, France: Longitudinal population-based study of randomly selected older adults (69%)<br><br>73.7 years	2089<br><br>461 all Dementia	5-15<br><br>Cases excluded if dementia detected within 3yr 'latent period'	1264<br>38%	Married (60.7%)<br>Widowed (32.5%)<br>Divorced (2.7%)<br>Single (4.2%)<br><br>Assessed for over 65s in 1988	Age; Sex; Edu; baseline cognition; positive affect; ADLs; Chronic diseases; quality and quantity of social network contact	Cox regression (with age as time-scale)	**Dementia**	Married<br>Widowed<br>Divorced<br>Single	HR 1<br>HR 0.88 (0.7, 1.1)<br>HR 0.94 (0.5, 1.7)<br>HR 1.29 (0.7, 2.1)	Not provided
		373 Alzheimer's Disease						**Alzheimer's Disease**<br><br>(Clinical assessment by neurologist using valid criteria)	Married<br>Widowed<br>Divorced<br>Single	HR 1<br>HR 0.92 (0.7, 1.1)<br>HR 0.88 (0.4, 1.7)<br>HR 1.36 (0.7, 2.3)	
Arai 2004	Hokkaido, Japan. Community-based prospective study<br><br>69 years	853<br><br>34	5	No data provided	Living with spouse 71%<br>Not living with spouse 29%<br>Living with others 15%<br><br>Assessed in 1998	Age; Sex	Mantel-Haentzel	**Dementia**<br><br>Clinical assessment based on algorithm	Living with spouse<br>Not living with spouse	RR 1<br>RR 2.0 (1.0, 5.0)	1<br>2.2
Bae 2014	Korean Longitudinal Study on Cognitive Aging and Dementia (71.6%)<br><br>71.7	359<br><br>45 all dementia	3.5	144<br>40%	Married 70.2%<br>Widowed 29.8%<br>Divorced 0%<br>Single 0%<br><br>Assessed in 2008	Age, sex	Cox regression	**Dementia**	Married<br>Widowed<br>Divorced<br>Single	HR 1<br>HR 1.79 (0.5, 6.5)<br>HR 0<br>HR 0	Not provided
		9 Alzheimer's Disease						**Alzheimer's Disease**<br><br>Clinical assessment by psychiatrist using valid criteria	Married<br>Widowed<br>Divorced<br>Single	HR 1<br>HR 4.40 (0.8, 24.7)<br>HR 0<br>HR 0	

Bickel 1994	Mannheim, Germany. Longitudinal population-based cohort of elderly persons in private households (82.1%)<br><br>73.8 years	331<br><br>34	7-8	12<br>4%	Married 42.4%<br>Widowed 47.5%<br>Divorced 3.8%<br>Single 6.4%<br><br>Assessed in 1992	Age, sex	Cox regression	**Dementia**<br><br>Clinical assessment by trained physicians based using valid criteria	Married<br>Widowed<br>Divorced<br>Single	HR 1<br>HR 1.59 (0.7, 3.5)<br>HR 3.17 (0.6, 16.4)<br>HR 2.90 (0.8, 10.5)	1<br>1.86<br>3.21<br>3.56
Fratiglioni 2000	Kungsholmen, Sweden: Longitudinal population-based study of community-dwelling people born before 1913 (76%)<br><br>81·5 years	1368<br><br>176	3	165<br>12%	Married 27.8%<br>Widowed 45.4%<br>Divorced 5.9%<br>Single 20.9%<br><br>Assessed in 1987	Age; Sex;<br>BL cognition	Cox regression	**Dementia**<br><br>Clinical assessment by 2 independent physicians using valid criteria	Married<br>Widowed<br>Divorced<br>Single	HR 1<br>HR 1.45 (0.9, 2.2)<br>HR 1.04 (0.5, 2.4)<br>HR 1.77 (1.1, 2.9)	1<br>1.6 (wid or div)<br>1.8
Håkansson 2009	CAIDE project. Longitudinal population-based study derived from random sampling in two regions in Eastern Finland (82-90%)<br><br>71.3 years	2000<br><br>44	20.9	511<br>2.7%	Married 80.1%<br>Widowed 7.8%<br>Divorced 4.4%<br>Single 7.8%<br><br>Mid-life – people aged 50.5 between 1972-87<br>Late life – people aged 71.3 in 1998	Age; Sex; Edu; ApoE; BMI; BP; Cholesterol; Occupation; Physical activity; Region; Smoking; Depression	Logistic regression	**Alzheimer's Disease**<br><br>Clinical assessment by expert board using valid criteria	*Mid-life marital status*<br>Married<br>Widowed<br>Single/divorced	 <br>OR 1<br>OR 2.52 (0.8, 7.7)<br>OR 1.78 (0.7, 4.9)	Not provided
									Mid-and late-life marital status change<br>Remained married<br>Became single<br>Remained single	 <br><br>OR 1<br>OR 1.60 (0.7, 3.8)<br>OR 2.83 (1.1, 7.4)	
Hatch 2013	Cache County Memory Study. Longitudinal population based study of all residents aged over 65, identified from Medicare records (90%)<br><br>74.6 years	5092<br><br>548 all dementia	12	1459<br>28.7%	Married 65.9%<br>Widowed 29.9%<br>Divorced 4.1%<br><br>Measured in 1995	Age; Sex; Occupation; ApoE	Cox regression	**Dementia**	Married<br>Widowed<br>Divorced	HR 1<br>HR 0.99 (0.81, 1.22)<br>HR 0.65 (0.37, 1.16)	1<br>1.75<br>0.67
		369 Alzheimer's disease						**Alzheimer's Disease**<br><br>(Clinical assessment by expert board using valid criteria)	Married<br>Widowed<br>Divorced	HR 1<br>HR 1.04 (0.82, 1.33)<br>HR 0.59 (0.28, 1.25)	1<br>2.05<br>0.64
Sundström 2014	Betula prospective cohort study, Umeå Sweden: Longitudinal population-based study	1677<br><br>354	8.6	32<br>2%	Married 57.6%<br>Widowed 14.2%<br>Divorced 5.7%<br>Single 32.6%	Age; Sex; Alcohol; mental illness; availability of a close friend;	Cox regression	**Dementia**<br><br>Clinical assessment by	Married<br>Widowed<br>Divorced<br>Single	1<br>HR 1.30 (1.0, 1.7)<br>HR 1.32 (0.9, 2.1)<br>HR 1.09 (0.6, 1.9)	 1.42<br>1.48<br>1.59

	derived from general population stratified by age and sex. (87%)<br><br>74·7 years				Assessed in 1993-5	parental status		2 independent physicians using valid criteria			
Sundström 2016	Linnaeus database, Sweden: Linked population data from healthcare and death records for entire population<br><br>69.4 years<br><br>(other group of people aged 50-64, mean age 56.1)	750129<br><br>25722	6	32065<br>1%	**Men**:<br>Married 68.1%<br>Widowed 3.5%<br>Divorced 15.0%<br>Single 13.5%<br>**Women**:<br>Married 61.8%<br>Widowed 13.1%<br>Divorced 17.0%<br>Single 8.2%<br><br>**Total:**<br>Married 64.9%<br>Widowed 8.4%<br>Divorced 16.0%<br>Single 10.8%<br><br>Assessed in 1997	Age; Sex; Parental status; Edu; Income; CVD	Cox regression	**Dementia**<br><br>Derived from clinical records or death certificates: Specificity 98% Sensitivity 55%	**All aged 65-74**<br>Married<br>Widowed<br>Divorced<br>Single	 HR 1<br>HR 1.12 (1.1, 1.2)<br>HR 1.42 (1.4, 1.5)<br>HR 1.23 (1.2, 1.3)	**(Age-adjusted)**<br>1<br>1.11<br>1.42<br>1.25
									**Men aged 65-74**<br>Married<br>Widowed<br>Divorced<br>Single	 HR 1<br>HR 1.10 (1.0, 1.2)<br>HR 1.47 (1.4, 1.6)<br>HR 1.29 (1.2, 1.4)	**(Age-adjusted)**<br>1<br>1.10<br>1.48<br>1.32
									**Women aged 65-74**<br>Married<br>Widowed<br>Divorced<br>Single	 HR 1<br>HR 1.10 (1.1, 1.4)<br>HR 1.36 (1.3, 1.4)<br>HR 1.16 (1.1, 1.3)	**(Age-adjusted)**<br>1<br>1.11<br>1.36<br>1.18
									**All aged 50-64**<br>Married<br>Widowed<br>Divorced<br>Single	 HR 1<br>HR 1.28 (1.1, 1.4)<br>HR 1.79 (1.7, 1.9)<br>HR 1.71 (1.6, 1.9)	Not provided
**CASE-CONTROL**				**Missing data**							
Beard 1992	Rochester, USA. Epidemiology Project. Cases selected from records of Mayo Clinic which delivered medical care to most residents.<br><br>80.4 years	241 cases<br>241 controls	N/A	0	Married 28.8%<br>Widowed 48.0%<br>Divorced 5.4%<br>Single 17.8%<br><br>Assessed at point of diagnosis (1975-79)	Matched by age and sex	Logistic regression	**Alzheimer's Disease**<br><br>Clinical diagnoses confirmed against valid criteria by psychiatrist.	Married<br>Widowed<br>Divorced<br>Single	OR 1<br>OR 1.10 (0.7, 1.7)<br>OR 1.25 (0.5, 2.9)<br>OR 1.07 (0.6, 1.8)	Not provided
									Men<br>Married<br>Widowed<br>Divorced<br>Single	 OR 1<br>OR 1.24 (0.8, 1.8)<br>OR 3.45 (0.9, 14.0)<br>OR 1.73 (0.3, 9.7)	Not provided
									Women<br>Married<br>Widowed<br>Divorced<br>Single	 OR 1<br>OR 0.98 (0.8, 1.2)<br>OR 0.77 (0.4, 1.4)<br>OR 0.94 (0.7, 1.2)	Not provided

Seidler<br>2003	Frankfurt, Germany. Cases selected from general practice registers: (77% agreed to participation)<br><br>Controls selected as random sample of population register >65 years (61%) AND sample from general practice register (90%)<br><br>Cases: 79.5 years<br>Controls: 75.4 years	195 cases<br>229<br>controls	N/A	29<br>6%	Married 78.5%<br>Widowed 11.1%<br>Divorced 3.8%<br>Single 6.6%<br><br>Derived in c2001 from interview with patient or next-of-kin based on marital status when 50yrs	Age; sex; edu; region; family history; smoking	Logistic regression	**Dementia**<br><br>Clinical diagnoses confirmed against valid criteria by psychiatrist.	*Status at 30yrs*<br>Married<br>Widowed<br>Divorced<br>Single	 *OR 1*<br>*OR 2.1 (0.7, 6.2)*<br>*OR 1.0 (0.2, 4.1)*<br>*OR 1.1 (0.6, 2.0)*	 1<br>2.50<br>1.14<br>1.23
									Status at 50yrs<br>Married<br>Widowed<br>Divorced<br>Single	 *OR 1*<br>*OR 1.2 (0.6, 2.3)*<br>*OR 0.6 (0.2, 1.8)*<br>*OR 1.1 0.5, 2.5)*	 1<br>1.69<br>0.58<br>1.36
									Status 10y earlier<br>Married<br>Widowed<br>Divorced<br>Single	 *OR 1*<br>*OR 1.0 (0.6, 1.6)*<br>*OR 0.5 (0.2, 1.7)*<br>*OR 1.7 (0.7, 4.2)*	 1<br>1.47<br>0.57<br>1.99
		108<br>Alzheimer's<br>disease						**Alzheimer's Disease**	*Status at 30yrs*<br>Married<br>Widowed<br>Divorced<br>Single	 *OR 1*<br>*OR 4.3 (1.4, 12.9)*<br>*OR 1.3 (0.2, 7.2)*<br>*OR 0.7 (0.3, 1.5)*	 1<br>4.14<br>1.04<br>0.99
		59 vascular<br>dementia						**Vascular dementia**	*Status at 30yrs*<br>Married<br>Widowed<br>Divorced<br>Single	 *OR 1*<br>*OR 1.7 (0.2, 14.9)*<br>*OR 1.5 (0.2, 13.7)*<br>*OR 1.7*	 1<br>0.73<br>0.92<br>1.43
**CROSS-SECTIONAL**				**Missing data**							
Correa<br>Ribeiro<br>2013	Rio de Janeiro, Brazil. Age and sex-stratified sample selected from clients of a private health-care plan: (98%)<br><br>78.2 years	683<br><br>115	N/A	108<br>12.5%	Married 41.6%<br>Widowed 40.8%<br>Divorced 7.5%<br>Single 10.1%<br><br>Derived from interview by researcher in 2009	Age; Sex; edu; Personal income	Log-binomial regression	**Dementia**<br><br>Clinical diagnoses by consensus panel on valid criteria	Married<br>Widowed<br>Divorced/separated<br>Single	RR 1<br>RR 1.43 (0.9, 2.3)<br>RR 0.31 (0.1, 2.0)<br>RR 0.81 (0.3, 2.1)	1<br>2.97<br>0.24<br>1.17
Fan 2015	Taiwan. Nationwide population-based cross-sectional study (36.5%)<br><br>75.7 years	10432<br><br>929	N/A	419<br>5.0%	Married 64.2%<br>Widowed 31.0%<br>Divorced/single 4.8%<br><br>Derived from researcher interview in 2012	Age; sex; edu; BMI; Hypertension; Diabetes; CVD; Smoking; alcohol; exercise; social engagement; sleep	Logistic regression	**Dementia**<br><br>Clinical diagnoses confirmed against valid criteria by psychiatrist.	Married<br>Widowed<br>Divorced/single	OR 1<br>OR 1.42 (1.2, 1.8)<br>OR 1.20 (0.7, 2.0)	1<br>2.65<br>1.67
Guaita 2015	Abbiategrasso, Italy. Survey of all residents	1321	N/A	2<br>0.2%	Married 67.1%<br>Widowed 24.6%	Age; Sex; Area of birth;	Logistic regression	**All dementia**<br>Clinical	Married<br>Widowed	RR 1<br>RR 1.17 (0.5, 2.7)	1<br>1.18

	aged 70-74 yr. (80.4%)<br><br>71.7 years	39 all dementia			Divorced 2.2%<br>Single 6.1%<br><br>Assessed in 2011	Occupation; Education		diagnoses confirmed against valid criteria by geriatrician	Divorced<br>Single	RR 0.87 (0.1, 7.2)<br>RR 2.52 (0.8, 7.8)	1.26<br>2.44
		15 Alzheimer's disease						**Alzheimer's Disease**	Married<br>Widowed<br>Divorced<br>Single	RR 1<br>RR 1.05 (0.3, 3.8)<br>RR 2.42 (0.3, 23.0)<br>RR 1.31 (0.2, 11.0)	1<br>1.18<br>3.09<br>1.18
		18 vascular dementia						**Vascular dementia**	Married<br>Widowed<br>Divorced<br>Single	RR 1<br>RR 1.8 (0.5, 6.2)<br>RR --<br>RR 5.63 (1.3, 23.8)	1<br>1.45<br>--<br>1.45
Zhang 2006	China:<br>prevalence study conducted across four different communities. (94%)<br><br>68.2 years	34807<br><br>732 Alzheimer's Disease	N/A	59<br>0.1%	Married 77.4%<br>Widowed 20.8%<br>Divorced/single 1.6%<br><br>Assessed in over 55s in 1997	Age; Sex; Edu; Rural/urban dwelling; ethnicity; occupation; age/region interaction; sex/education interaction	Logistic regression	**Alzheimer's Disease**	Married<br>Widowed<br>Divorced/Single	OR 1<br>OR 1.4 (1.1, 1.7)<br>OR 2.0 (0.8, 5.0)	1<br>5.2<br>2.3
		295 vascular dementia						**Vascular Dementia**<br><br>Consensus panel diagnosis after 3-phase assessment	Married<br>Widowed<br>Divorced/Single	OR 1<br>OR 0.6 (0.5, 0.9)<br>OR 1.0 (0.4, 2.4)	1<br>1.1<br>0.9

Key: ADLs = Activities of daily living; BMI = body mass index; CVD = cardiovascular disease; Edu = education; HR = Hazard ratio; OR = Odds ratio; RR = Risk ratio

Notes: Shaded results are those which have been provided on request by study authors. *Italicised results* are those which we calculated from study data e.g. when confidence intervals were not provided.

Supplementary table 3. Rating criteria for quality of included studies and results from quality rating.

* Indicates a point for methodological quality

Supplementary table 3a - COHORT STUDIES

Selection

1) Representativeness of the exposed cohort
    a. truly representative of the average person over 65 years in the community with initial response rate over 70% *
    b. selected group of users e.g. nurses, volunteers
    c. no description of the derivation of the cohort
2) Selection of the non-exposed cohort
    a. drawn from the same community as the exposed cohort *
    b. drawn from a different source
    c. no description of the derivation of the non-exposed cohort
3) Ascertainment of exposure
    a. secure record (e.g. public records) *
    b. structured questionnaire with details on timing of potential changes of marital status *
    c. written self-report
    d. no description
4) Demonstration that outcome of interest was not present at start of study
    a. yes *
    b. no

Comparability

5) Comparability of cohorts on the basis of the design or analysis (2 * possible)
    a. As well as **age and sex**, the study controls for **Education or baseline cognition***
    b. Study additionally controls for a **measure of physical illness** AND **socio-economic status ***
    c. Only adjusts for **age and sex**

Outcome

6) Assessment of outcome (*dementia*)
    a. Systematic blind assessment using standard diagnostic criteria *
    b. Record linkage
    c. Self- or carer- report
    d. no description
7) Was follow-up long enough for outcomes to occur
    a. At least 5 years *
    b. no
8) Adequacy of follow up of cohorts
    a. complete follow up - all subjects accounted for *
    b. Less than <30 % lost to follow up *
    c. follow up rate < 70% (select an adequate %) and no description of those lost
    d. no statement

		1<br>*	2<br>*	3<br>*	4<br>*	5<br>* / **	6<br>*	7<br>*	8<br>*	Total
**1**	**Amieva**	c	*	d	*	*	*	*	c	**5**
**2**	**Arai**	c	*	d	*	c	c	*	d	**3**
**3**	**Bae**	b	*	d	*	c	*	b	c	**3**
**4**	**Bickel**	b	*	d	*	c	*	*	*	**5**
**5**	**Fratiglioni**	*	*	d	*	*	*	b	*	**6**
**6**	**Håkansson**	*	*	d	*	**	*	*	*	**8**
**7**	**Hatch**	b	*	*	*	**	*	*	*	**5**
**8**	**Sundström 2014**	*	*	d	*	*	*	*	*	**7**
**9**	**Sundström 2016**	*	*	*	*	**	b	*	*	**8**

Supplementary table 3b - <u>CASE-CONTROL STUDIES</u>

Selection

1) Is the case definition (*dementia diagnosis*) adequate?
    a. yes, with independent validation *****
    b. Record linkage
    c. no description
2) Representativeness of the cases
    a. consecutive or obviously representative series of cases *****
    b. potential for selection biases or not stated
3) Selection of Controls
    a. community controls *****
    b. hospital controls
    c. no description
4) Definition of Controls
    a. no history of disease (endpoint) *****
    b. no description of source

Comparability

e. Comparability of cases and controls on the basis of the design or analysis (2 * possible)
    a. As well as **age and sex**, the study controls for **Education or baseline cognition***
    b. study also controls for a **measure of physical illness** AND **socio-economic status ***
    c. Only adjusts for **age and sex**

Exposure

f. Ascertainment of exposure
    a. secure record (eg public records) *****
    b. structured questionnaire with details on timing of potential changes of marital status AND independent verification (notes OR informant) *
    c. interview not blinded to case/control status
    d. written self-report or medical record only
    e. no description
g. Same method of ascertainment for cases and controls
    a. yes *****
    b. no
h. Non-Response rate
    a. same rate for both groups *****
    b. non respondents described
    c. rate different and no designation

		**1**<br>*	**2**<br>*	**3**<br>*	**4**<br>*	**5**<br>* / **	**6**<br>*	**7**<br>*	**8**<br>*	**Total**
**1**	**Beard**	b	b	*	b	-	c	*	*	**3**
**2**	**Seidler**	b	b	b	b	*	c	b	b	**1**

Supplementary table 3c - CROSS-SECTIONAL STUDIES

Selection

1) Were the criteria for inclusion in the sample clearly defined?
   a. truly representative of the average person over 65 years in the community *
   b. selected group of users eg volunteers
   c. no description of the derivation of the sample
2) Was the initial response rate reported?
   a. Reported and > 70% *
   b. Reported and < 70%
   c. Not reported

Measurements

3) Ascertainment of exposure
   a. secure record (eg public records) *****
   b. structured questionnaire with details on timing of potential changes of marital status AND independent verification (notes OR informant) *
   c. interview not blinded to dementia status
   d. written self-report or medical record only
   e. no description
4) Assessment of Dementia
   a. Systematic blind assessment using standard diagnostic criteria *
   b. Record linkage *
   c. Self- or carer- report
   d. no description

Comparability

5) Comparability of cases and controls on the basis of the design or analysis (2 * possible)
   a. As well as **age** and **sex**, the study controls for **Education** or **baseline cognition***
   b. study controls for a measure of **physical illness** AND **socio-economic status** *
   c. Only adjusts for **age and sex**

		**1**<br>*****	**2**<br>*****	**3**<br>*****	**4**<br>*****	**5**<br>*** / ****	
**1**	**Correa-Ribeiro**	b	*	c	*	*	**3**
**2**	**Fan**	*	*	c	*	*	**4**
**3**	**Guaita**	*	*	c	*	*	**4**
**4**	**Zhang**	*	*	e	*	*	**4**

Supplementary figure 1. Begg's funnel plots for main meta-analyses showing risk for publication bias in published studies.

Widowed v married

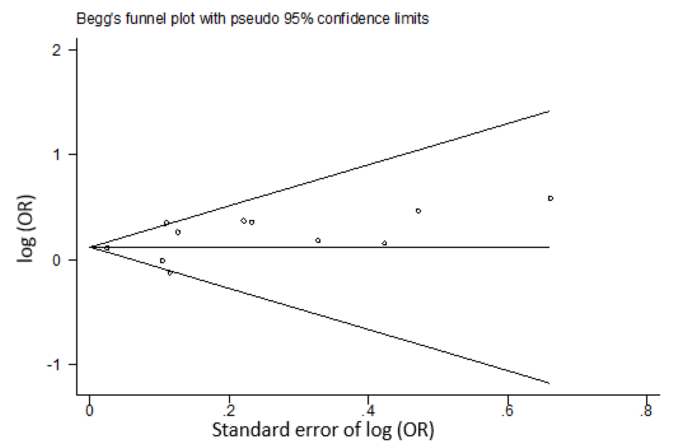

Divorced v married

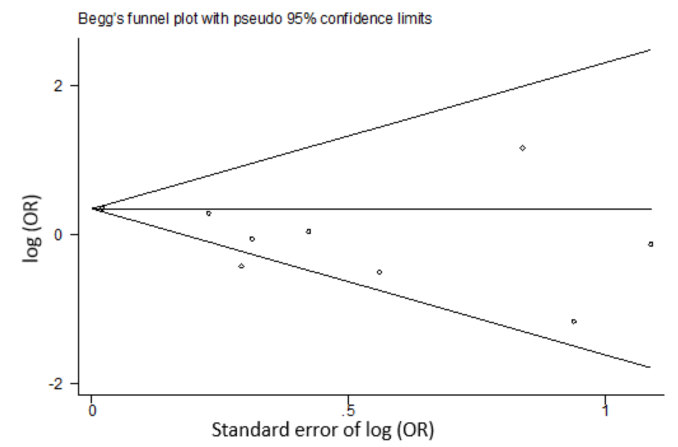

Lifelong single v married

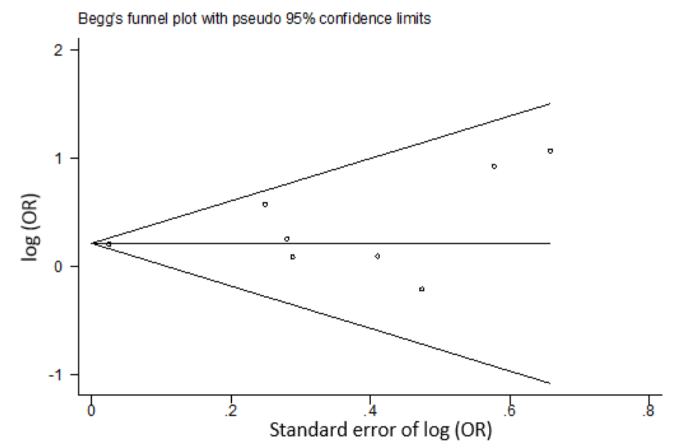

Supplement: Supplementary material 1 [file jnnp-2017-316274supp001.pdf]
